# Metabolic engineering of *Corynebacterium glutamicum* for the production of *cis*, *cis*-muconic acid from lignin

**DOI:** 10.1186/s12934-018-0963-2

**Published:** 2018-07-20

**Authors:** Judith Becker, Martin Kuhl, Michael Kohlstedt, Sören Starck, Christoph Wittmann

**Affiliations:** Institute of Systems Biotechnology, Saarland University, Campus A1.5, Saarbrücken, Germany

**Keywords:** Lignin, Bio-plastic, Adipic acid, Terephthalic acid, Catechol dioxygenase, Muconate cycloisomerase, Metabolic engineering, Aromatics

## Abstract

**Background:**

*Cis*, *cis*-muconic acid (MA) is a dicarboxylic acid of recognized industrial value. It provides direct access to adipic acid and terephthalic acid, prominent monomers of commercial plastics.

**Results:**

In the present work, we engineered the soil bacterium *Corynebacterium glutamicum* into a stable genome-based cell factory for high-level production of bio-based MA from aromatics and lignin hydrolysates. The elimination of muconate cycloisomerase (*catB*) in the catechol branch of the β-ketoadipate pathway provided a mutant, which accumulated MA at 100% molar yield from catechol, phenol, and benzoic acid, using glucose as additional growth substrate. The production of MA was optimized by constitutive overexpression of *catA*, which increased the activity of the encoded catechol 1,2-dioxygenase, forming MA from catechol, tenfold. Intracellular levels of catechol were more than 30-fold lower than extracellular levels, minimizing toxicity, but still saturating the high affinity CatA enzyme. In a fed-batch process, the created strain *C. glutamicum* MA-2 accumulated 85 g L^−1^ MA from catechol in 60 h and achieved a maximum volumetric productivity of 2.4 g L^−1^ h^−1^. The strain was furthermore used to demonstrate the production of MA from lignin in a cascade process. Following hydrothermal depolymerization of softwood lignin into small aromatics, the MA-2 strain accumulated 1.8 g L^−1^ MA from the obtained hydrolysate.

**Conclusions:**

Our findings open the door to valorize lignin, the second most abundant polymer on earth, by metabolically engineered *C. glutamicum* for industrial production of MA and potentially other chemicals.

**Electronic supplementary material:**

The online version of this article (10.1186/s12934-018-0963-2) contains supplementary material, which is available to authorized users.

## Background

*Cis*, *cis*-muconic acid (MA) is a di-unsaturated dicarboxylic acid of recognized industrial value [1]. MA is easily hydrogenated into adipic acid, a widely applied building block of commercial nylons and polyurethanes. In addition, MA can be utilized as the starting material for making terephthalic acid, which is one of the two constituent monomers of the high-demand plastic polymer polyethylene terephthalate (PET) [2]. Adipic acid and terephthalic acid also have wide applications in the cosmetic, pharmaceutical and food industries [1]. The increasing prices and diminishing resources for petroleum are drivers to produce plastics through environmentally friendly fermentation from renewables, instead of using petro chemistry [3], explaining the obvious interest in bio-based MA.

Different microorganisms, such as *Pseudomonas putida* KT2440 [4], *Amycolatopsis species* ATCC 39116 [15], and *E. coli* [5] have been engineered to produce MA either via biosynthesis from glucose [6–8] and glycerol [2] or via biotransformation from aromatics [9–11]. The latter is particularly advantageous, because it requires only a few biochemical reactions and offers molar yields up to 100% [4]. So far, MA production has been demonstrated for different aromatics, including benzoate [12, 13], toluene [14], catechol [5, 13], phenol [13], *p*-coumarate [7], and guaiacol [15], reaching titers up to 65 g L^−1^ [13] and productivities up to 4.9 g L^−1^ h^−1^ [5]. More importantly, strains which produce MA from aromatics offer the use of lignin hydrolysates as starting material [13, 16, 17]. Lignin displays the second most abundant polymer in nature and can be processed into mixtures of small aromatics, such as catechol and phenol [13] through thermochemical [18–20] and biological de-polymerization [21]. To date, lignin is strongly underutilized: 98% of the polymer is simply burned, so that it represents a considerable source of renewable carbon [22]. In contrast to aromatics-based biotransformation, de-novo biosynthesis of MA from glucose to glycerol is less efficient. So far, MA titer (5 g L^−1^) and productivity (0.03 g L^−1^ h^−1^) have remained rather low [2, 7, 8].

**Fig. 1:**
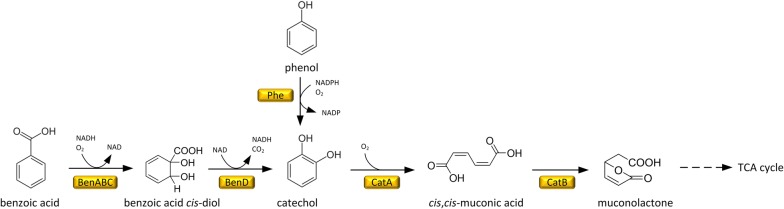
Catabolic pathway for aromatic compounds in *C. glutamicum*. The displayed reactions comprise the degradation routes for benzoic acid, catechol, and phenol. *BenABC* benzoate 1,2-diogygenase, *BenD* benzoate *cis*-diol dehydrogenase, *CatA* catechol 1,2-dioxygenase, *CatB* muconate cycloisomerase, *Phe* phenol 2-monooxygenase

Recently, our group metabolically engineered the soil bacterium *Corynebacterium glutamicum* for the production of lysine [23], ectoine [24], diaminopentane [25] and aminovalerate [26], using different sugars. Interestingly, *C. glutamicum* degrades a rich spectrum of aromatic compounds via the catechol branch of the β-ketoadipate pathway, involving MA as a pathway intermediate (Fig. 1) [27–30]. The β-ketoadipate pathway is the major pathway for lignin-derived aromatic compounds assimilation [27], suggesting that engineered strains of *C. glutamicum* could produce MA from lignin-based aromatics.

Here, we describe stepwise metabolic engineering of *C. glutamicum* to convert the aromatics benzoic acid, catechol, and phenol into MA. Biotransformation of catechol into MA was additionally carried out in a fed-batch process to evaluate the performance of the engineered strain. Furthermore, we were able to establish MA production directly from a lignin hydrolysate.

## Results

### Deletion of muconate-cycloisomerase enables MA production from small aromatics

In order to block the catabolization of small aromatics at the level of MA, the *catB* gene encoding muconate-cycloisomerase, was deleted from the genome of the wildtype *C. glutamicum* ATCC 13032. Clones obtained after the second homologous recombination were analyzed for the desired modification. The deletion was found successful on basis of the shortened PCR product of 1050 bp (Additional file 1: Figure S1). The wild type control exhibited a fragment size of 1750 bp, representing the native gene. The resulting mutant *C. glutamicum* ATCC130232 Δ*catB* was designated *C. glutamicum* MA-1. In contrast to the wild type, the MA-1 strain was no longer able to grow on the aromatic compounds benzoic acid, catechol, and phenol as sole carbon source (data not shown). All aromatics remained at the initially added level. In contrast, aromatics consumption and also growth of MA-1 was enabled by adding small amounts of glucose. Under these conditions, the MA-1 strain completely converted 5 mM phenol, 10 mM catechol, and 20 mM benzoic acid, respectively, within 24 h. MA was found in the culture supernatant for each of the aromatics tested. The molar conversion yield of the aromatics into MA was close to 100% (Table 1, Additional file 1: Figure S2A). However, when higher concentrations of aromatics were supplied, the conversion was incomplete after 24 h (data not shown), a result probably due to the inhibitory effects of these substrates.

**Table 1:** Kinetics and stoichiometry of *cis*, *cis*-muconic acid (MA) production of *Corynebacterium glutamicum* MA-1 from benzoic acid, catechol or phenol, and of *Corynebacterium glutamicum* MA-2 from catechol

Strain substrate	MA-1 benzoic acid	MA-1 catechol	MA-1 phenol	MA-1* catechol	MA-2 catechol
*q*_*MA*_ [mmol g^−1^ h^−1^]	3.3 ± 0.1	0.2 ± 0.0	0.1 ± 0.0	3.5 ± 0.1	5.2 ± 0.4
*q*_*Glc*_ [mmol g^−1^ h^−1^]	0.5 ± 0.0	0.3 ± 0.0	0.6 ± 0.0	0.4 ± 0.0	0.7 ± 0.0
*Y*_*X/Glc*_ [g g^−1^]	0.46 ± 0.01	0.38 ± 0.01	0.48 ± 0.01	0.40 ± 0.02	0.40 ± 0.02
*Y*_*MA/Aro*_ [mol mol^−1^]	1.00 ± 0.02	0.99 ± 0.02	0.95 ± 0.09	1.00 ± 0.01	1.00 ± 0.01

The data comprise the specific glucose uptake rate (*q*_*Glc*_), the specific MA production rate (*q*_*MA*_), the biomass yield (*Y*_*X/Glc*_*),* and the MA yield (*Y*_*MA/Aro*_) and represent mean values and standard deviations from three biological replicates. The culture of the MA-1* strain additionally contained benzoate as inducer

### *C. glutamicum* MA-1 shows high tolerance to small aromatics

A set of growth experiments was conducted to study potential inhibitory effects of the biotransformation substrates on growth. The mutant MA-1 exhibited a high tolerance and grew up to high levels of catechol (30 mM), phenol (30 mM), and benzoic acid (80 mM), the highest concentrations tested. The non-charged aromatics catechol and phenol were more toxic and caused a stronger growth inhibition than the acid (Additional file 1: Figure S3). The strain exhibited a 50% growth reduction at a concentration (K_I_) of 22.5 mM catechol **(**Additional file 1: Figure S2B).

### The efficiency of MA production depends on the aromatic substrate

**Fig. 2:**
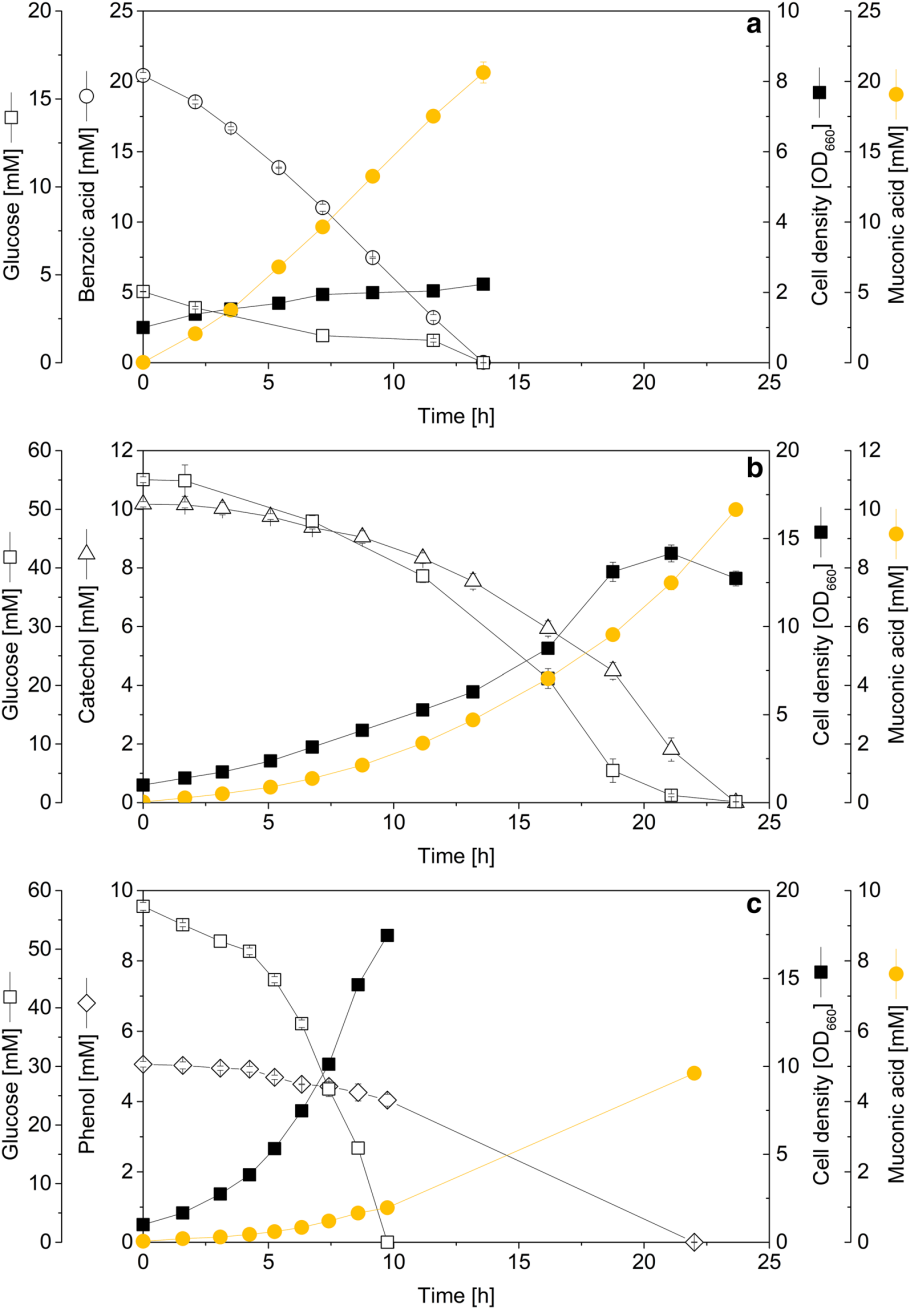
Kinetics and stoichiometry of *cis*–*cis*-muconic acid (MA) production, using the first generation producer *Corynebacterium glutamicum* MA-1. The aromatics benzoic acid (**a**), catechol (**b**) and phenol (**c**) were used for production. In addition, glucose was added as growth substrate. The data represent the culture profiles until the depletion of the aromatic. The full culture profiles are given in the supplement (Additional file 1: Figure S4). The concentration of glucose was corrected to represent the amount consumed during this phase. The data comprise mean values and deviations from three biological replicates

Although the conversion into MA was complete in all cases, the cells differed strongly in their substrate preference (Fig. 2a–c). The MA-1 strain consumed benzoic acid (20 mM) immediately and accumulated 20 mM MA within only 14 h. During this phase, benzoic acid was preferred over glucose: the cells assimilated only small amounts of the sugar, showed minor growth and rather formed MA in a growth-decoupled biotransformation. At the time point of benzoic acid depletion, most of the glucose was still present (Additional file 1: Figure S4). In contrast, catechol was co-consumed with glucose, and the MA production was growth associated (Fig. 2b). Consequently, the MA accumulation rate increased with increasing cell concentration. The conversion of 10 mM catechol took about 20 h, whereby catechol was converted into MA with a molar yield of 100% (Table 1). Phenol was metabolized slower (Fig. 2c), and cells preferred glucose. Overall, a molar MA yield from phenol of nearly 100% was achieved (Table 1). Regarding cellular growth, the cultures on benzoic acid and on phenol exhibited similar biomass yields, whereas the catechol-grown cells were somewhat less efficient and exhibited a lower biomass yield.

### The induction level of catechol-1,2-dioxygenase is responsible for the MA production performance

Catechol-1,2-dioxygenase (CatA), the MA forming enzyme in *C. glutamicum,* was studied to explore its impact on the substrate-dependence in more detail. A culture on glucose, but without aromatics added, revealed a CatA activity of 30 mU mg^−1^ (Additional file 1: Figure S5A). Interestingly, the addition of either catechol or phenol to the growth medium did not result in any change of the CatA activity. In fact, CatA was still expressed at a basal level. In contrast, the cells showed 16-fold higher CatA activity, when cultivated on benzoic acid (490 mU mg^−1^). The crude extract of benzoic acid-grown cells was used to assess the kinetics of the CatA enzyme, in particular the substrate affinity of the enzyme. The reaction rates, obtained at different catechol levels were fitted to a Michaelis–Menten type kinetics (Additional file 1: Figure S5B). The K_M_-value for catechol, enabling operation of the enzyme at 50% of its maximum rate, was 2.3 µM. Hence, the enzyme has a high affinity, enabling efficient conversions already at low level of the substrate. Obviously, the poor production performance on catechol and phenol seemed due to a limited expression of the key enzyme CatA.

### Targeted overexpression of catechol-1,2-dioxygenase decouples the enzyme from native induction and enables faster MA production from catechol

**Fig. 3:**
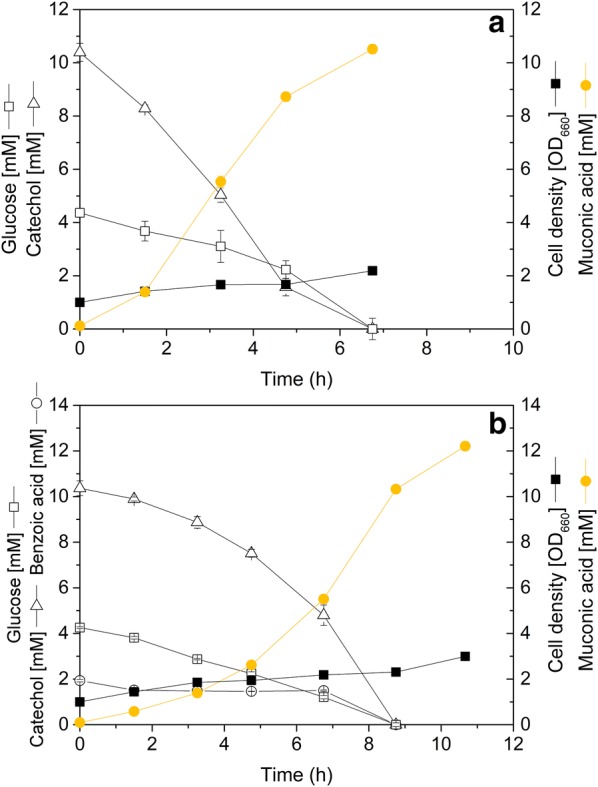
Culture profile of the second generation producer MA-2, using 10 mM catechol for production (**a**), and of the first generation producer MA-1, using 10 mM catechol for production plus 2 mM benzoic acid for induction of *catA* expression (**b**). In addition, glucose was added as growth substrate. The data represent the culture profiles until the depletion of the aromatic. The full culture profiles are given in the supplement (Additional file 1: Figure S7). The concentration of glucose was corrected to represent the amount consumed during this phase. The data comprise mean values and deviations from three biological replicates

To overcome the bottleneck at the level of CatA, the *catA* gene was overexpressed, using the strong constitutive *tuf* promoter. The genetic modification was verified by PCR (Additional file 1: Figure S6) and sequencing. The obtained strain *C. glutamicum ΔcatB P*_*tuf*_*catA*, was designated *C. glutamicum* MA-2. To investigate the effect of the promoter exchange, the newly constructed mutant was analyzed for its CatA activity (Additional file 1: Figure S5C). When the cells were grown on either benzoic acid, catechol or phenol, CatA was generally expressed at high activity (330 mU mg^−1^), independent from the substrate used. In comparison to the native control, the *tuf* promoter, enabled tenfold higher CatA levels on the relevant substrates, catechol and on phenol. For benzoic acid, the enzyme activity was 30% lower than that of the native induction system. The constitutive overexpression of *catA* in the strain MA-2 enabled a faster conversion of catechol to MA (Fig. 3a), corresponding to a specific MA production rate of 5.2 mmol g^−1^ h^−1^ (0.7 g g^−1^ h^−1^) (Table 1). The rate was 25-fold higher than that of the parent, un-induced strain (Table 1) and 1.5-fold higher than that of the parent strain, additionally induced with benzoic acid (Fig. 3b, Table 1). The molar MA yield on catechol was 100%. Cells showed minor growth, until the entire catechol was converted and co-consumed the aromatic together with small amounts of glucose. The cells contained intracellular levels of 330 ± 42 µmol g^−1^ catechol and 200 ± 18 mol g^−1^ MA during the mid-phase of the production process.

### Pulse-wise feeding of catechol reveals excellent process robustness of *C. glutamicum* MA-2

**Fig. 4:**
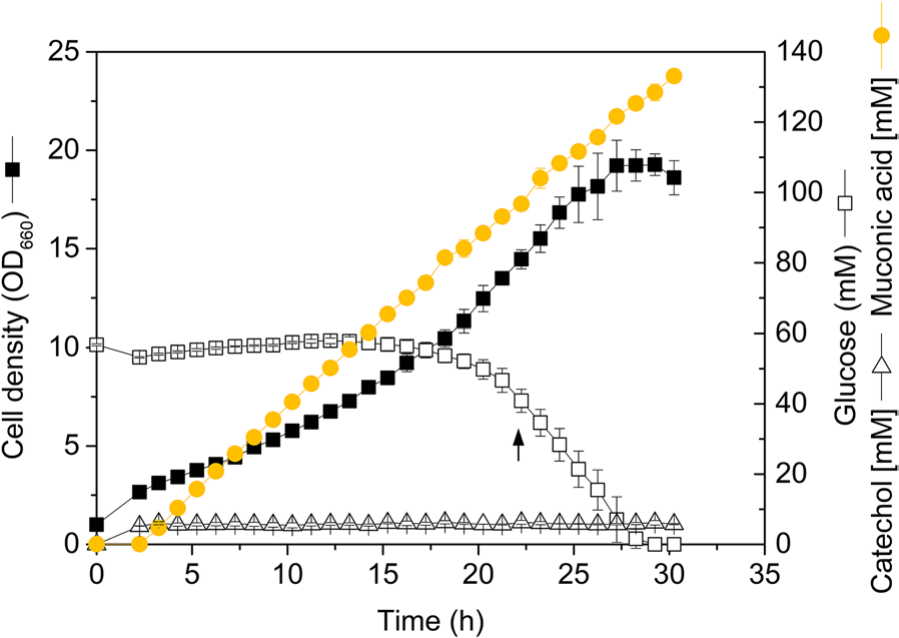
Production of c*is*–*cis*-muconic acid (MA) with feeding of catechol. The production was conducted in a shake flask. Metabolically engineered *Corynebacterium glutamicum* MA-2 was grown on glucose minimal medium for the first 2 h. Then, the feeding was started. Every hour, pulses with 5 mM catechol and 0.5 g L^−1^ glucose, were added. In addition, the pH was controlled above 7.0 by manual control, adding appropriate volumes of 10 mM NaOH. The black arrow indicates the time point, when the glucose feed was stopped. The data represent mean values and standard deviations from three biological replicates

A fed-batch operated shake flask culture should aim for higher MA titers, using pulse-wise feeding of catechol to avoid toxic levels. *C. glutamicum* MA-2 was grown on glucose for the first 2 h and then fed every hour with catechol (Fig. 4). It was interesting to note that the specific growth rate immediately dropped to about 0.08 h^−1^, when the first feed pulse was added. The cells kept growing at this reduced rate. Throughout the entire cultivation, the added aromatic was completely converted into the target product. Catechol did not accumulate at any stage of the process. After 30 h, the cells achieved a final MA titer of 19 g L^−1^ (133 mM) at a molar product yield from catechol of 100%. The high production efficiency was maintained until the end of the cultivation. After about 24 h, the glucose addition was intentionally stopped to study the impact of the sugar. Even when glucose was depleted, the cells still maintained MA accumulation.

### *C. glutamicum* MA-2 sets a benchmark in high-level MA production

**Fig. 5:**
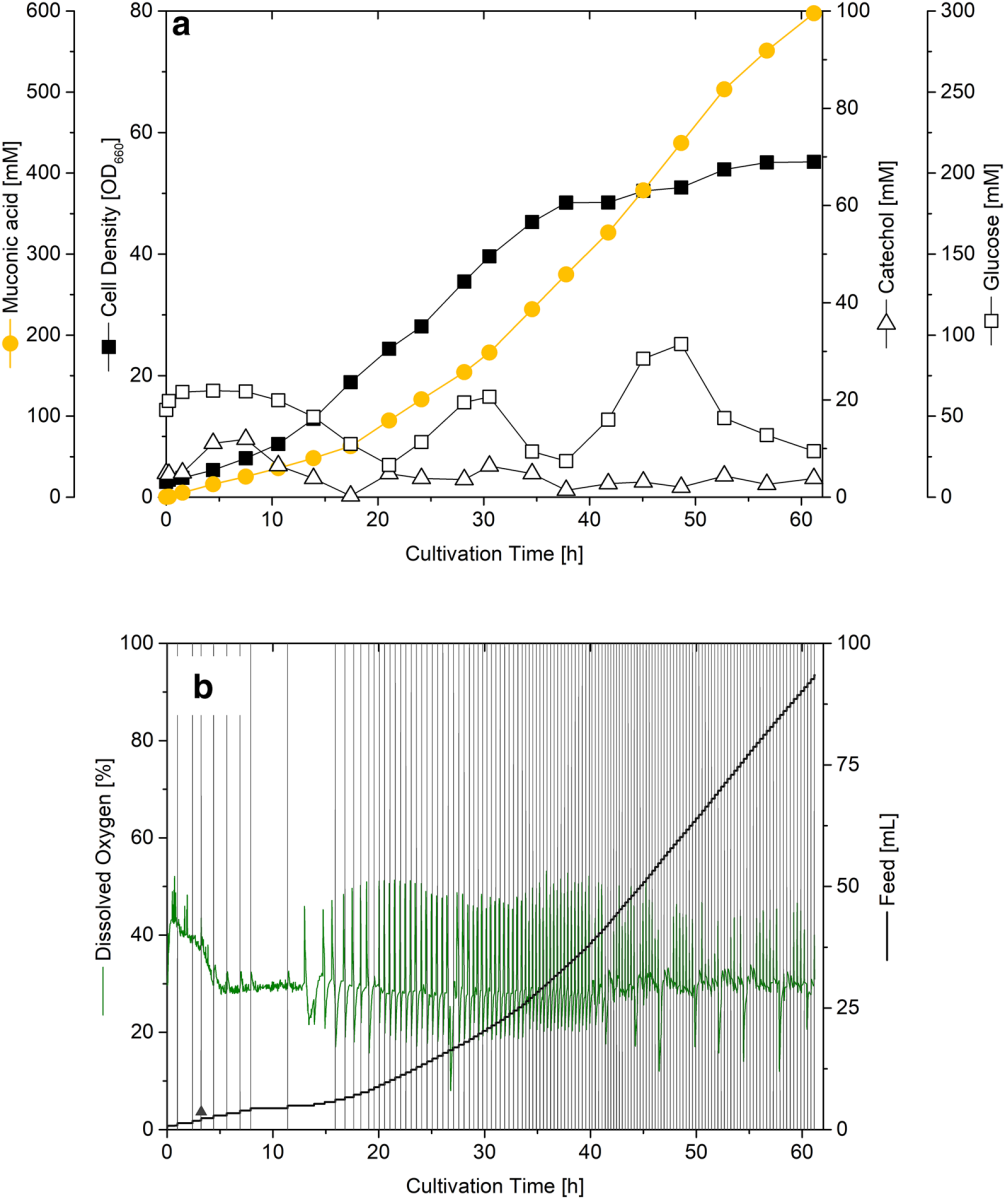
Fed-batch production of c*is*–*cis*-muconic acid (MA) from catechol by metabolically engineered *Corynebacterium glutamicum* MA-2. Substrate consumption, growth and MA formation (**a**). Pulse-wise feeding of catechol (**b**). Glucose was added continuously to maintain the glucose level in the range between about 5–15 g L^−1^ (**a**). The vertical lines represent individual catechol feed pulses (**b**). The feed frequency was variably adjusted, depending on the signal of dissolved oxygen, which precisely indicated the time point of catechol depletion. As example, feeding was halted once during the initial phase, corresponding to transient catechol accumulation and was accelerated later in response to the faster conversion. The data represent mean values from two replicates. The fermentation data, specifying the MA level and the biomass concentration in g L^−1^ (Additional file 1: Figure S8A), the volumetric productivity (Additional file 1: Figure S8B) and the different yields (Additional file 1: Figure S8C), are given in the supplement

The production performance of *C. glutamicum* MA-2 was next investigated in a fed-batch process, using a lean medium, containing only catechol, sugar, and salts. The MA production started immediately and reached a titer of 85 g L^−1^ within 60 h (Fig. 5a). The cell concentration increased from initially 1 g L^−1^ to about 14 g L^−1^ after 34 h, roughly half of the total process time. During this phase, glucose was co-consumed with catechol and utilized for growth at a reduced rate. At the end of this initial phase, about 30 g L^−1^ MA was formed. Subsequently, the cells switched to an almost exclusive production mode for the rest of the process. They converted catechol at an even higher rate into MA, and formed additionally more than 50 g L^−1^ of the product within 25 h, but did not grow anymore and consumed only limited amounts of glucose. The space–time-yield increased continuously during the process and reached a maximum value of 2.4 g L^−1^ h^−1^ (Additional file 1: Figure S8). During the entire process, the strain exhibited a constant molar MA yield from catechol of 100%. The carbon-based product yield, i.e. the amount of MA formed from glucose plus catechol, differed in the different process phases (Additional file 1: Figure S8). In the first process phase, substantial glucose was consumed for biomass formation concomitantly with catechol consumption for MA formation, in sum resulting in a carbon yield of 0.49 mol mol^−1^. In the following phase, the carbon yield increased up to 0.81 mol mol^−1^, as growth and glucose consumption dropped (Additional file 1: Figure S8). Regarding the process control, the on-line signal for dissolved oxygen (DO) served as excellent trigger for the addition of new catechol, which directly resulted from the underlying pathway stoichiometry (Fig. 1). Each time when catechol was depleted and the oxygen-dependent reaction of CatA was accordingly halted, the DO signal sharply increased (Fig. 5b). Likewise, also the pH value sensitively indicated the physiology of the cells and dropped during each interval, as long as MA was formed. The established feed addition allowed a tight control of catechol in the broth at low level. An exception was a phase in the early process stage, when the feed exceeded the capacity of the cells and the catechol level transiently raised to more than 10 mM in concentration. However, the cells could cope with this process environment and quickly degraded catechol to levels below 5 mM, as long as the feed addition was halted.

### Cascaded chemical and biochemical process to convert lignin into MA

**Fig. 6:**
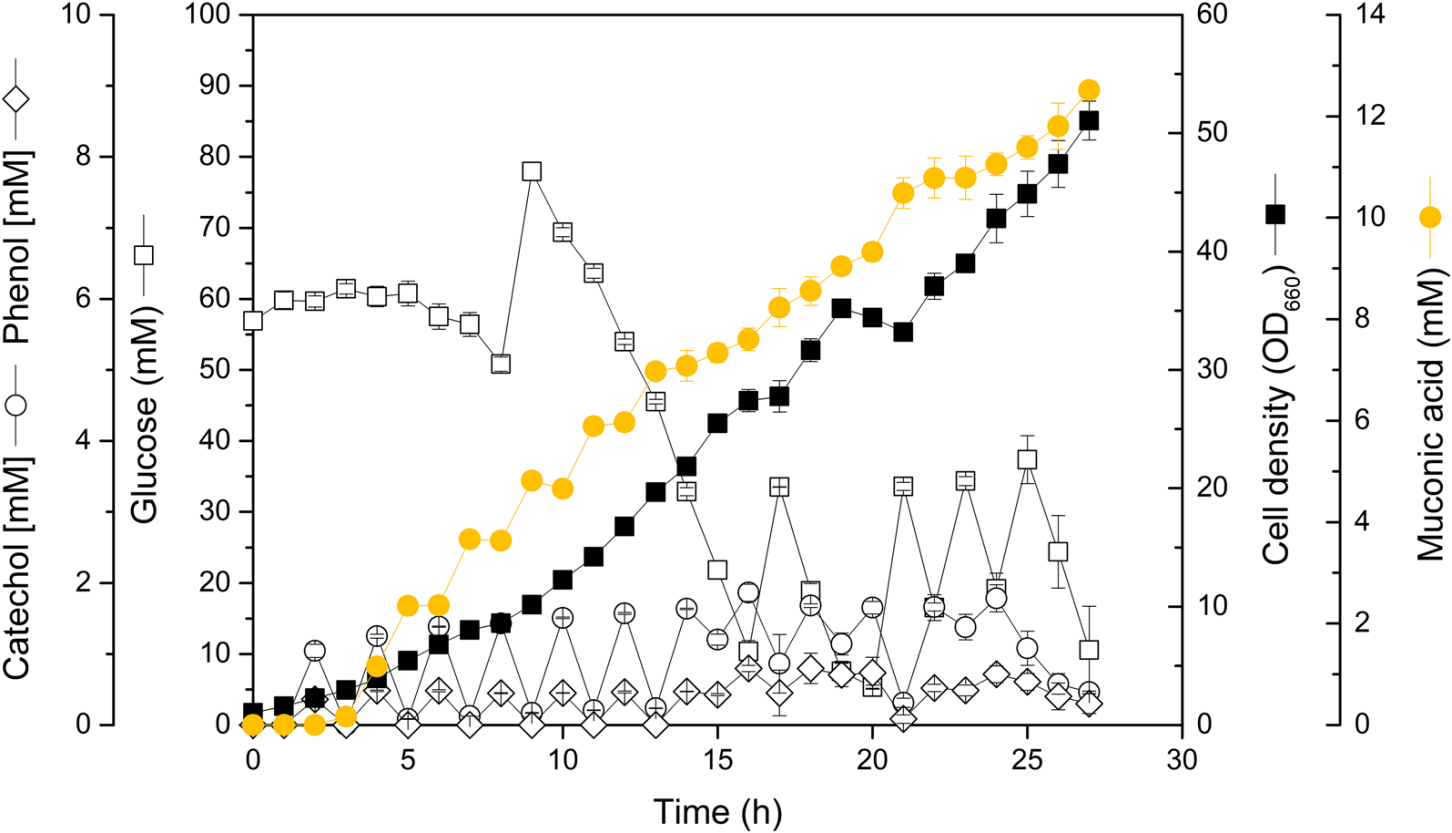
Production of *cis*, *cis*-muconic (MA) from softwood lignin hydrolysate, using the engineered producer *Corynebacterium glutamicum* MA-2 with glucose as a growth substrate and pulsed feeding of the hydrolysate. Feeding was stopped after 12 pulses due to limited availability of the hydrolysate. The data represent mean values and deviations from three replicates

The cascaded process started with the depolymerization of lignin as a first step. For this purpose, softwood lignin was hydrolyzed into defined aromatic monomers, using hydrothermal treatment in supercritical water. The incubation in the pressure reactor resulted in the accumulation of catechol and phenol in the liquid phase, which was then concentrated (Table 2). The engineered MA-2 strain successfully converted the lignin hydrolysate, added pulse-wise to the culture, into MA (Fig. 6). Within 27 h, an MA titer of 12.5 mM (1.8 g L^−1^) was achieved. The molar MA yield on the two aromatics was close to 100%.

**Table 2:** Hydrothermal treatment of softwood lignin

Compound	Catechol	Phenol	Total
Yield [% w/w]	5.7 ± 0.1	3.9 ± 0.1	9.6 ± 0.3
Content (mM)	101	25	126

The data comprise the yield of catechol and phenol from the hydrothermal treatment, representing three replicates (upper line) and their content in the concentrate, collected from three hydrothermal runs and subsequently enriched (lower line)

## Discussion

### MA production in engineered *C. glutamicum* from small aromatics comes at attractive titer, yield and productivity

MA is a chemical of recognized industrial value and accessible from aromatics through a highly attractive route [4, 7, 17, 22]. From the metabolic pathway perspective, the catechol node displays the centerpiece for this type of production: catechol displays the terminal pathway intermediate of MA biosynthesis independent from the aromatics used and is also a most relevant ingredient, generated during lignin pre-processing [13].

Here we show that engineered of *C. glutamicum* strains efficiently utilize small aromatics and convert them into MA. On basis of titer, productivity and yield, performance indicators of industrial fermentation processes, the created producer *C. glutamicum* MA-2 has remarkable properties. The MA titer of 85 g L^−1^ surpasses the highest achievements of the past decades by almost 50% [1]. Even until recently, and using sophisticated approaches such as sensor-based evolution [31], carefully-established co-cultures [2], new production hosts [2, 15, 31–33] or novel biosynthetic pathways [34], MA titers typically remained at rather low level. The observed volumetric productivity (2.4 g L^−1^ h^−1^) competes well with other biotransformation processes, which have been described to produce MA [5, 14], aminovalerate [35, 36], and cadaverine [37]. Furthermore, this productivity lies in the range of advanced fermentation processes for the production of chemicals using *C. glutamicum* [23, 38]. Regarding stoichiometry, the aromatics were converted into MA at a molar yield of 100%. Although one might expect such a high conversion efficiency from the underlying pathway stoichiometry (Fig. 1), molar yields of biotransformations can be substantially lower, being the case for aminovalerate from lysine (69% molar yield) [35] or for MA from benzoic acid (47% molar yield) [39].

### *C. glutamicum* MA-2 offers key features of successful MA producer

Four features have been recently claimed as key to success in MA bio-production from aromatics: (i) a high CatA activity, (ii) elimination of CatB activity, (iii) robustness to aromatics, and (iv) active export of muconate [1]. All these features are provided by *C. glutamicum* MA-2. Firstly, the integrated synthetic control of CatA expression provides high activity of the enzyme, independent of any induction requirement. Moreover, the high affinity of the enzyme to its substrate catechol enables an efficient conversion, even for the low levels of intracellular catechol. Secondly, the producer strain, lacking the *catB* gene, lost the ability of the wild type to use benzoic acid, catechol, and phenol as sole carbon source, confirming that the connection between the β-ketoadipate pathway and the central carbon metabolism was fully disrupted and that no other degradation routes for the three aromatics exist in *C. glutamicum* [27]. Thirdly, the strain tolerated about three-fold higher catechol levels than *P. putida*, one of the best performing aromatics degrading microbes known [13]. Even process-related fluctuations of the aromatics level did not disturb the metabolic activity of *C. glutamicum* MA-2, so that the conversion could be operated in a reproducible and robust manner. Although the toxic catechol [40] was continuously present in the millimolar range, cells maintained high production efficiency for the entire process duration of 72 h. One explanation for the obvious robustness could be the low intracellular level of catechol, which was more than 30-fold lower than that outside of the cells and likely benefited from several natural properties of *C. glutamicum*. Its CatA enzyme has a high affinity for catechol, so that even the low levels present exceeded the K_M_ value of CatA about 65-fold and therefore enabled efficient conversion. In addition, *C. glutamicum* possesses an outer membrane, although staining Gram-positive, which forms an efficient permeability barrier that contributes to its high resistance to drugs [41]. The high tolerance of *C. glutamicum* is also a major advantage for the desired use of aromatics from lignin [42]. We produced 1.8 g L^−1^ of MA from depolymerized softwood lignin, demonstrating that the MA-2 strain, growing well during the incubation, can tolerate and convert such a complex and inhibitory substrate mixture. The MA titer was limited by the amount of lignin hydrolysate, but not by an impaired fitness of the producer strain, so that higher values appear feasible, given larger scale lignin pre-processing. Finally yet importantly, *C. glutamicum* obviously operates an active muconate exporter. MA was found in the supernatant, whereby the product was secreted against a concentration gradient, as the intracellular MA level of 100 µM was about 50-fold lower than the concentration outside of the cell.

### Synthetic control of *catA* expression is crucial for efficient MA production from small aromatics

The activity of catechol-1,2-dixoygenase, encoded by *catA* [43], is a metabolic bottleneck for MA production from aromatics as it dictates the conversion speed [13]. The stimulating effect of benzoic acid on CatA activity and MA production observed in this work, nicely matched with previous models, describing the activation of the *ben* and the *cat* operon in *C. glutamicum* by benzoic acid [27]. Unfavorably, the naturally un-induced pathway provided only basal levels of CatA activity on the two relevant lignin-based aromatics, catechol and phenol. Constitutive overexpression of *catA*, using the homologous *tuf* promoter [44] overcame this bottleneck. Interestingly, for *P. putida*, use of the native promoter of *catA* expression was more favorable for MA production from catechol than the use of a constitutive promoter [13] despite the complex regulation of the *P. putida cat*-operon [19].

### Future strategies for enhancing MA production in *C. glutamicum*

The relation of (i) the maximum possible flux of an enzyme and (ii) the real flux catalyzed provides a quantitative insight into pathway efficiency and possible limitations [38]. For the MA production pathway it is interesting to determine this relation at the level of the key enzyme catechol-1,2-dioxygenase (CatA). The maximum possible CatA flux was 10 mmol g^−1^ h^−1^ for the best producer, on basis of the measured enzyme activity and a cellular protein content of 50% [45]. The activity is about twice as high as the observed flux of 5.2 mmol g^−1^ h^−1^ providing a pathway efficiency of about 50%. Although this value appears surprisingly high, the cells did not fully exploit their theoretical potential, leaving space for future rounds of metabolic engineering. An interesting candidate to be engineered in the future might be the export of MA out of the cell. The product was obviously secreted against a concentration gradient, as the intracellular MA level of about 100 µM was much lower than that outside of the cell at the time point of sampling (about 5 mM). Accordingly, MA is likely exported by an active transport process, similar to other dicarboxylic acids in the microbe [46], which appears also reasonable given the highly charged nature of the molecule. The MA exporter in *C. glutamicum* is, to date, unknown, suggesting further studies to identify this protein [47]. In addition, the energy and redox metabolism might play a role to further enhance the cellular vitality, which would require a more demanding systems-wide approach of metabolic engineering [3]. Moreover, an optimized process operation might provide higher MA titers from catechol to other aromatics. The dissolved oxygen signal, fluctuating during the process in response to the production (Fig. 1), could be exploited for automated feeding of catechol, which might circumvent inhibitory effects, eventually being imposed by the substrate.

## Conclusion

In this work, we established *C. glutamicum* for production of the industrial platform chemical *cis*, *cis*-muconic acid (MA) from aromatics through genetically stable genome manipulation. Most biotechnology processes, using cell factories of *C. glutamicum*, rely on sugars [48], often competing with human nutrition. In this regard, the use of aromatic compounds for bio-production with *C. glutamicum* is regarded promising [49]. The route of interest, engineered in this work for the production of MA, is the catechol branch of the β-ketoadipate pathway. It is encoded in the genome of *C. glutamicum* and has been explored to great detail by a set of careful biochemical studies [27–29, 43, 50]. Naturally, this pathway is used for the assimilation of catechol, benzoic acid, and phenol, which all converge at the level of catechol. *C. glutamicum* MA-2, the newly created cell factory, enabled the efficient use of the two lignin aromatics catechol and phenol. Both compounds are formed during lignin depolymerization and display relevant raw materials for industrial production [51]. In particular, catechol is highly important for future lignin valorization. It is a central hub of aromatic catabolism: multiple degradation pathways converge at this node, making it a key intermediate to funnel further aromatics into bio-production [17]. We demonstrated this for phenol, which is typically contained in lignin-based materials [22]. Future work could aim to implement guaiacol utilizing pathways into the microbe [15]. In addition, the introduction of protocatechuate decarboxylase [7] would connect the entire protocatechuate branch, encoded in *C. glutamicum* [27], with the catechol node and potentially enable the conversion of further additional monomers such as coumarate. In addition to such metabolic engineering strategies, more bioprocess engineering is needed to upgrade MA production from the proof-of-concept level, demonstrated in our study towards industrially attractive efficiency. This might include further research to select most suitable wood types and lignin depolymerization strategies and provide hydrolysates with high bioavailability and low toxicity [18, 21, 51]. Moreover, an optimization of the fermentation process itself appears promising to drive MA production from lignin hydrolysates further. A pulse-wise hydrolysate feeding at an excess of sugar could enable high cell densities at minimized toxicity [13]. In addition, process strategies, which automatically couple the feeding of aromatics for MA production to the control of the pH value or dissolved oxygen level, seem interesting concepts to be tested [4].

## Methods

### Microorganisms and plasmids

*Corynebacterium glutamicum* ATCC 13032 was obtained from the American Type Culture Collection (Manassas, VA, USA). *Escherichia coli* stellar (Clontech Laboratories, Mountain View, CA, USA) and NM522 (Invitrogen, Carlsbad, CA, USA) were used for cloning purposes. *E. coli* NM522 harbors the plasmid pTc, that expresses a *C. glutamicum* specific DNA-methyltransferase [52]. This is needed, because *C. glutamicum* cells identify and degrade non methylated DNA. For genomic modification of *C. glutamicum*, the integrative, non-replicating plasmid pClik int sacB was used [44].

### Molecular design and genetic engineering

For molecular strain, plasmid and primer design, the Clone Manager Professional 9 (Sci-Ed Software, Denver, USA) was used. The genetic construct for deletion of the *catB* gene (NCgl2318) in the genome of *C. glutamicum* comprised a DNA fragment, lacking 703 bp of the target gene and 500 bp-sized flanking regions as homologous recombination sites. For overexpression of the *catA* gene (NCgl2319) the genetic construct consisted of a 200 bp fragment of the promoter of the structural *tuf* gene (NCgl0480) and 500 bp-sized flanking regions as homologous recombination sites. All DNA fragments were amplified by PCR (2 × Phusion Flash PCR Master Mix, Thermo Scientific, Waltham, MA, USA and peQSTAR, PEQLAB Biotechnology GmbH, Erlangen, Germany) from genomic DNA of *C. glutamicum* ATCC13032 with sequence specific primers (Table 3). DNA fragment and vector assembly was carried out by the method of Gibson [53]. Prior to the assembly, the vector was linearized via restriction with *Bam*HI (FastDigest, Thermo Fisher Scientific). Vector amplification in the *E. coli* strains Stellar and NM522, purification of plasmid DNA, and plasmid transformation into *E. coli* and *C. glutamicum* strains were performed as described previously [54]. PCR and sequence analysis (GATC Biotech AG, Konstanz, Germany) were used for plasmid and strain validation.

**Table 3:** Description of primers that were used in the present work for genome-based deletion of the *catB* gene (NCgl2318) and integration of the *tuf*-promoter for overexpression of the *catA* gene (NCgl2319) in *Corynebacterium glutamicum*

Name	Sequence	Use	AT [°C]
Δ*catB* TS1 FW	5′-TTAACAATTGGGATCCTCTAGACCCACTCCAGAGCATTGGGGTGTTT-3′	Amplification of TS1; TS1 FW+ overlap to pClik, TS 1 RV+ overlap to TS2 FW	55
Δ*catB* TS1 RV	5′-AGGTCCAATCGGCACCGCAGCAT-3′		
Δ*catB* TS2 FW	5′-GGATGCTGCGGTGCCGATTGGACCCTCAAGCGCCTTCATGGTCTCAAC-3′	Amplification of TS2; TS2 FW+ overlap to TS1 RV, TS 2 RV+ overlap to pClik	57
Δ*catB* TS2 RV	5′-GCAGCCCGCTAGCGATTTAAATCCCTGGGCACCCATGGCGCCCAG-3′		
P_*ef*-*tu*_-*catA*-TS1 FW	5′-AATTGGGATCCTCTAGACCCACGATTCCCTCTTCAGGGCG-3′	Amplification of TS1; TS1 FW+ overlap to pClik, TS1 RV+ overlap to P_*ef*-*tu*_	61
P_*ef*-*tu*_-*catA*-TS1 RV	5′-CATTCGCAGGGTAACGGCCAGTCATACTCCTAGAGACTTTGGGGG-3′		
P_ef-t*u*_-*catA*-P_*ef*–*tu*_ FW	5′-AAAGTCTCTAGGAGTATGACTGGCCGTTACCCTGCGAATG-3′	Amplification of P_*ef*–*tu*_; P_*ef*–*tu*_ FW+ overlap to TS1 RV, P_*ef*–*tu*_ RV+ overlap to TS2 FW	62
P_*ef*-*tu*_-*catA*-P_*ef*–*tu*_ RV	5′-ATCTGTTCAGCTGAAGTCATTGTATGTCCTCCTGGACTTCGTGG-3′		
P_*ef*-*tu*_-*catA*-TS2 FW	5′-GAAGTCCAGGAGGACATACAATGACTTCAGCTGAACAGATCG-3′	Amplification of TS2; TS2 FW+ overlap to P_*ef*-*tu*_, TS2 RV + overlap to pClik	57
P_*ef*-*tu*_-*catA*-TS2 RV	5′-CCGCTAGCGATTTAAATCCCTAGTATCCGTCCTCATCTGCG-3′		
PR154	5′-ATTGTCTGTTGTGCCCAGTCATAG-3′	Validation of vector pClik int *sacB* (including respective insert)	53
PR318	5′-AATAATAGTGAACGGCAGGT-3′		

*AT* annealing temperature

### Tolerance testing

*Corynebacterium glutamicum* was grown at 1 mL-scale in a micro bioreactor (BioLector I, m2plabs, Baesweiler, Germany), using 48-well flower plates (MTP-48-B, m2plabs, Baesweiler, Germany). The incubation at different levels of catechol, phenol, and benzoic acid was conducted at 1300 rpm and 30 °C. All cultures were carried out as biological triplicate.

### Batch production of MA from aromatics in shake flasks

*Corynebacterium glutamicum* was grown in baffled shake flasks with 10% filling volume at 30 °C and 230 rpm on an orbital shaker (Multitron, Infors AG, Bottmingen, Switzerland, 5 cm shaking diameter). The cultivation procedure involved one pre-culture in complex medium (37 g L^−1^ BHI) followed by another pre-cultivation and then by the main cultivation, both in minimal medium [55], which contained the following salts and vitamins per liter: NaCl, 0.055 g CaCl_2_·H_2_O, 0.2 g MgSO_4_·7H_2_O, 15 g (NH_4_)_2_SO_4_, 24.98 g K_2_HPO_4_, 7.7 g KH_2_PO_4_, 20 mg FeSO_4_·7 H_2_O, 0.5 mg biotin, 1 mg thiamin·HCl, 30 mg 3,4-dihydroxybenzoic acid and 10 mL of a 100× trace element solution [56]. The medium was additionally supplemented with different amounts of benzoic acid, catechol, phenol, and glucose from filter sterilized stocks either alone or in mixtures as described below. The different medium ingredients were combined at room temperature freshly before use. The pH was kept constant at 7.0 ± 0.2 by manual addition of 2 M NaOH. All cultures were conducted as biological triplicate.

### Hydrothermal lignin depolymerization and production of MA from the obtained lignin hydrolysate

Hydrothermal conversion of lignin from pine (IndulinAT, S3Chemicals, Bad Oeynhausen, Germany) was conducted as described previously [13]. The obtained hydrolysate was clarified by centrifugation (10,000×*g*, 5 min, and room temperature) and subsequently concentrated (Vacuum Concentrator RVC 2-33 IR, Christ Gefriertrocknungsanlagen, Osterode, Germany) to be used as raw material for the production. For this purpose, *C. glutamicum* was grown in minimal medium in baffled shake flasks as described above. The medium was additionally supplemented with glucose. About 8 h after the inoculation, the lignin hydrolysate was added as a pulse. The pH was kept constant at 7.0 ± 0.2 by manual addition of 2 M NaOH. The cultures were conducted as biological triplicate.

### Fed-batch production of MA from catechol in shake flasks

The pre-culture scheme was as described above. The main culture was grown in minimal glucose medium, which was supplemented pulse-wise with glucose and with catechol. The catechol feed rate of 5 mM h^−1^ was chosen on basis of the estimated specific productivity of the cells in batch culture (Table 2) The feed comprised 500 µL of a catechol stock (1 M) and 500 µL of a glucose stock (100 g L^−1^). Considering the sample amount of 1 mL, taken every hour, the overall culture volume (100 mL) remained constant, so that each pulse increased the catechol concentration by 5 mM. The pH was maintained constant at 7.0 ± 0.2 by manual addition of 2 M NaOH. The cultures were conducted as biological triplicate.

### Fed-batch production of MA in a stirred tank bioreactor

The production performance of the optimized producer *C. glutamicum* MA-2 was evaluated in a fed-batch process in 1000 mL bioreactors (SR0700ODLS, DASGIP AG). The cultivation temperature was kept constant at 30 °C via the CWD4 bio-block (DASGIP AG, Jülich, Germany). The pH and the pO_2_ level were monitored online with a pH electrode (Mettler Toledo, Giessen, Germany) and a pO_2_ electrode (Hamilton, Höchst, Germany). The pH was kept constant at 7.0 ± 0.1 by automated addition of 12 M NaOH (MP8 pump system, Eppendorf, Hamburg, Germany). The dissolved oxygen level was maintained at saturation above 30% by variation of the stirrer speed and the aeration rate. The initial batch of 300 mL minimal medium with 10 g L^−1^ glucose and 5 mM catechol was inoculated with cells as described above. Catechol was added pulse-wise from a concentrated feed (4 M), using the signal of the dissolved oxygen probe as a trigger. In addition, a glucose feed was given continuously. The feed rate was re-adjusted, when needed, to maintain the level of glucose in a range of about 5–15 g L^−1^. The feed contained per liter: 450 g glucose, 70 g (NH_4_)_2_SO_4_, 1 g NaCl, 0.055 g CaCl_2_·H_2_O, 0.2 g MgSO_4_·7H_2_O, 15 g (NH_4_)_2_SO_4_, 24.98 g K_2_HPO_4_, 7.7 g KH_2_PO_4_, 20 mg FeSO_4_·7 H_2_O, 0.5 mg biotin, 1 mg thiamin·HCl, 30 mg 3,4-dihydroxybenzoic acid, and 100 mL of a 100× trace element solution [56]. Data acquisition and process operations were controlled by the DASGIP control software (DASGIP AG). The production process was conducted as duplicate.

### Extraction of intracellular metabolites

Two mL of exponentially growing cells were harvested by vacuum filtration (cellulose nitrate membrane filters, 0.2 µm pore size, 47 mm, Sartorius, Göttingen, Germany). The filter was washed with 15 mL of 2.5% NaCl, matching the ionic strength of the medium, and was then transferred into a plastic cup, containing 2 mL of boiling deionized water [57]. The sample was incubated in a water bath at 100 °C for 15 min and then chilled on ice. The extract was transferred into a fresh vial and clarified from cell debris (5 min, 13,000× g, and 4 °C).

### Substrate and product quantification

Catechol, phenol, benzoic acid, and MA were quantified by HPLC (Agilent 1200 Series, Agilent Technologies, Waldbronn, Germany), including separation at 25 °C on a reversed phase column (Nucleodur E100/3 C18 Isis 3 µm, Macherey–Nagel, Weilmünster, Germany) with a gradient of water (0.0035% H_3_PO_4_) and acetonitrile as mobile phase at a flow rate of 1 mL min^−1^. A diode array detector was used for detection of MA (260 nm) and the aromatics (210 nm). The quantification of glucose was carried out by isocratic HPLC (Agilent 1260 Infinity Series, Waldbronn, Germany). The separation was conducted on an Aminex HPX-87H column (300 × 7.8 mm; Bio-Rad, Munich, Germany) at 55 °C. As mobile phase, 3.5 mM H_2_SO_4_ was used at a flow rate of 0.8 mL min^−1^. The refraction index was used for detection. The concentration of cell dry mass (CDM) was calculated from the measured optical density (OD_660_) using a correlation factor of CDM (g L^−1^) = 0.32 × OD_660_ [26]. The exact monitoring of the cell concentration during the intracellular metabolite sampling and the above correlation allowed to relate the obtained metabolite levels to the CDM (µmol g_CDM_^−1^). The correlation between the cytoplasmic volume and the CDM (1.95 mL g_CDM_^−1^) was used to express intracellular concentrations in mM [58].

### Determination of enzyme activity

Crude cell extracts were prepared from exponentially growing cells by mechanical cell disruption. Cell harvest was carried out as previously described [25]. Aliquots of 1 mL cell suspension were transferred into FastPrep-24 vials (MP Biomedicals, Illkirch-Graffenstaden, France), containing silica beads (Ø 0.1 mm). Cell disruption was carried out in 2 × 30 s cycles at 5000 rpm (Precellys-24, Peqlab, Hannover, Germany), including a 5 min cooling pause on ice. The extract was also chilled on ice after the disruption. Cell debris was then removed by centrifugation (5 min, 13,000×*g*, and 4 °C). The activity of catechol-1,2-dioxygenase (CatA) was assayed in Tris–HCl buffer (100 mM, pH 8.2, 0.75 mM DTT). For this purpose, 900 µL buffer was mixed with 50 µL catechol (1 mM, pH 7.0) and 50 µL crude cell extract. The formation of MA (ε = 16.8 mL µM^−1^ cm^−1^) was monitored via the change in absorbance at 260 nm [59]. Negative controls were conducted without the addition of crude cell extract and catechol, respectively. The substrate affinity of the enzyme was determined by varying the concentration of catechol. The kinetic parameters of the enzyme were obtained by fitting the experimental data to the Michaelis–Menten type kinetic equation (OriginLab, Northhampton, MA, USA). The protein content in the crude cell extract was quantified, using bovine serum albumin as standard (Pierce BCA Protein Assay Kit, Thermo Fisher Scientific).

## Additional file

**Additional file 1: Figure S1.** Confirmation of the deletion of the *catB* gene in *Corynebacterium glutamicum* ATCC 13032, using colony PCR. To this end, *C. glutamicum* ATCC 13032, had been transformed with the integrative plasmid pClik int *sacB* Δ*catB*, followed by recombination and selection. The primers Δ*catB* TS1 FW and Δ*catB* TS2 RV were used for the PCR (Table 1). The positive clone, indicated by the white arrow, revealed the small fragment, size expected for the deletion. It was designated *C. glutamicum* LIMA-1. M, 1 kb DNA ladder; 1, blank; 2, positive clone; WT, wild type. **Figure S2.** Growth and *cis*–*cis*-muconic acid (MA) production from small aromatics, using *Corynebacterium glutamicum* MA-1. The yield for MA on benzoic acid (20 mM), catechol (10 mM), and phenol (5 mM) was obtained from measurement of substrates and product at the beginning and after 24 h of incubation (A). The tolerance to catechol was obtained from cell growth measurement (B). All data represent mean values and standard deviations from three biological replicates. **Figure S3.** Tolerance of *Corynebacterium glutamicum* MA-1 against benzoic acid (A), catechol (B), and phenol (C). In addition, glucose was added as growth substrate. The final cell concentration was measured after 24 h of cultivation. The data represent mean values and standard deviations from three biological replicates. **Figure S4.** Kinetics and stoichiometry of *cis*–*cis*-muconic acid (MA) production, using the first generation producer *Corynebacterium glutamicum* MA-1. The aromatics benzoic acid (A), catechol (B) and phenol (C) were used for production. In addition, glucose was added as growth substrate. The data represent mean values and standard deviations from three biological replicates. **Figure S5.** Characteristics of catechol-1,2-dioxygenase (CatA) in *Corynebacterium glutamicum*, grown on different aromatics. The data comprise the specific enzyme activity of the first generation producer *C. glutamicum* MA-1 (A), the kinetics of the enzyme with a fit of the experimental data from benzoate-grown cells to a Michaelis–Menten type kinetics (B), and the specific enzyme activity of the second generation producer *C. glutamicum* MA-2 (C). The data represent mean values and standard deviations from three biological replicates. **Figure S6.** Confirmation of overexpression of the *cat*A gene under control of the *tuf* promoter in *Corynebacterium glutamicum* MA-2, using colony PCR. To this end, *C. glutamicum* MA-1 had been transformed with the integrative plasmid pClik int *sacB P*_*tuf*_*catA*, followed by recombination and selection. The primers P_*ef*-*tu*_-*catA* TS1 FW and P_*ef*-*tu*_-*catA* TS2 RV were used for the PCR (Table 1). The positive clone, indicated by the white arrow, revealed the increased fragment size, expected for the promoter exchange. M, 1 kb DNA ladder; 1, positive clone; WT, wild type. **Figure S7.** Kinetics and stoichiometry of *cis*–*cis*-muconic acid (MA) production, using the second generation producer *Corynebacterium glutamicum* MA-2 on catechol (A) and the first generation producer *C. glutamicum* MA-1 on catechol and benzoic acid (B). Glucose was added as growth substrate. The data represent mean values and standard deviations from three biological replicates. **Figure S8.** Fed-batch production of c*is*–*cis*-muconic acid (MA) from catechol by metabolically engineered *Corynebacterium glutamicum* MA-2. Substrate consumption, growth and MA formation (A). Volumetric productivity (B). Yields for MA from catechol, and for MA from catechol plus glucose (C). Pulse-wise feeding of catechol (D). Glucose was added continuously to maintain the glucose level in the range between about 5 to 15 g L^−1^ (A). The vertical lines represent individual catechol feed pulses (D). The feed frequency was variably adjusted, depending on the signal of dissolved oxygen, which precisely indicated the time point of catechol depletion. As example, feeding was halted once during the initial phase, corresponding to transient catechol accumulation and was accelerated later in response to the faster conversion. The data represent mean values from two replicates.

## Abbreviations

*catA*: gene encoding for catechol-1,2-dioxygenase
*catB*: gene encoding for muconate-cycloisomerase
CDM: cell dry mass
MA: *cis*, *cis*-muconic acid
MCS: multiple cloning site
ORI: origin of replication
PCR: polymerase chain reaction
*sacB*: encoding gene of levansucrase from *Bacillus subtilis*
*tuf*: encoding gene of elongation factor tu
