# Supplementary material for: Metabolic engineering of Corynebacterium glutamicum for the production of cis, cis-muconic acid from lignin
Source: Microb Cell Fact. 2018 Jul 20;17:115. doi: 10.1186/s12934-018-0963-2 (PMC6054733; doi:10.1186/s12934-018-0963-2)
Supplement: Supplementary file 1 — Additional file 1: Figure S1. Confirmation of the deletion of the catB gene in Corynebacterium glutamicum ATCC 13032, using colony PCR. To this end, C. glutamicum ATCC 13032, had been transformed with the integrative plasmid pClik int sacB ΔcatB, followed by recombination and selection. The primers ΔcatB TS1 FW and ΔcatB TS2 RV were used for the PCR (Table 1). The positive clone, indicated by the white arrow, revealed the small fragment, size expected for the deletion. It was designated C. glutamicum LIMA-1. M, 1 kb DNA ladder; 1, blank; 2, positive clone; WT, wild type. Figure S2. Growth and cis–cis-muconic acid (MA) production from small aromatics, using Corynebacterium glutamicum MA-1. The yield for MA on benzoic acid (20 mM), catechol (10 mM), and phenol (5 mM) was obtained from measurement of substrates and product at the beginning and after 24 h of incubation (A). The tolerance to catechol was obtained from cell growth measurement (B). All data represent mean values and standard deviations from three biological replicates. Figure S3. Tolerance of Corynebacterium glutamicum MA-1 against benzoic acid (A), catechol (B), and phenol (C). In addition, glucose was added as growth substrate. The final cell concentration was measured after 24 h of cultivation. The data represent mean values and standard deviations from three biological replicates. Figure S4. Kinetics and stoichiometry of cis–cis-muconic acid (MA) production, using the first generation producer Corynebacterium glutamicum MA-1. The aromatics benzoic acid (A), catechol (B) and phenol (C) were used for production. In addition, glucose was added as growth substrate. The data represent mean values and standard deviations from three biological replicates. Figure S5. Characteristics of catechol-1,2-dioxygenase (CatA) in Corynebacterium glutamicum, grown on different aromatics. The data comprise the specific enzyme activity of the first generation producer C. glutamicum MA-1 (A), the kinetics of the enzyme with [file 12934_2018_963_MOESM1_ESM.docx]

**Metabolic engineering of *Corynebacterium glutamicum* for the production of *cis*,*cis*-muconic acid from lignin**

Revised version for publication in *Microbial Cell Factories*

Judith Becker^≠^, Martin Kuhl^≠^, Michael Kohlstedt, Sören Starck, and Christoph Wittmann^#^

Institute of Systems Biotechnology, Campus A1.5, Saarland University, Saarbrücken, Germany

^≠^ These authors contributed equally

^#^Corresponding author: Phone: +49 681 302 71970, FAX: +49 681 302 71972, e-mail: [christoph.wittmann@uni-saarland.de](mailto:christoph.wittmann@uni-saarland.de)


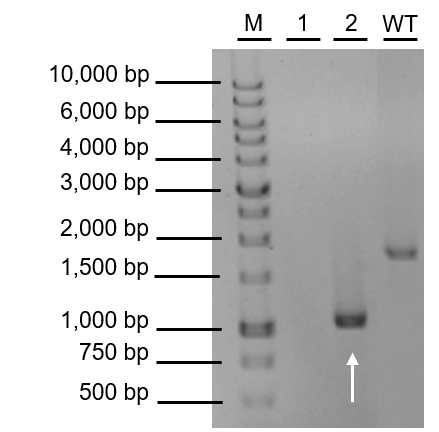


Figure S1: Confirmation of the deletion of the *catB* gene in *Corynebacterium glutamicum* ATCC 13032, using colony PCR. To this end, *C. glutamicum* ATCC 13032, had been transformed with the integrative plasmid pClik int *sacB* Δ*catB*, followed by recombination and selection. The primers Δ*catB* TS1 FW and Δ*catB* TS2 RV were used for the PCR (Table 1). The positive clone, indicated by the white arrow, revealed the small fragment, size expected for the deletion. It was designated *C. glutamicum* LIMA-1. M, 1 kb DNA ladder; 1, blank; 2, positive clone; WT, wild type.

**
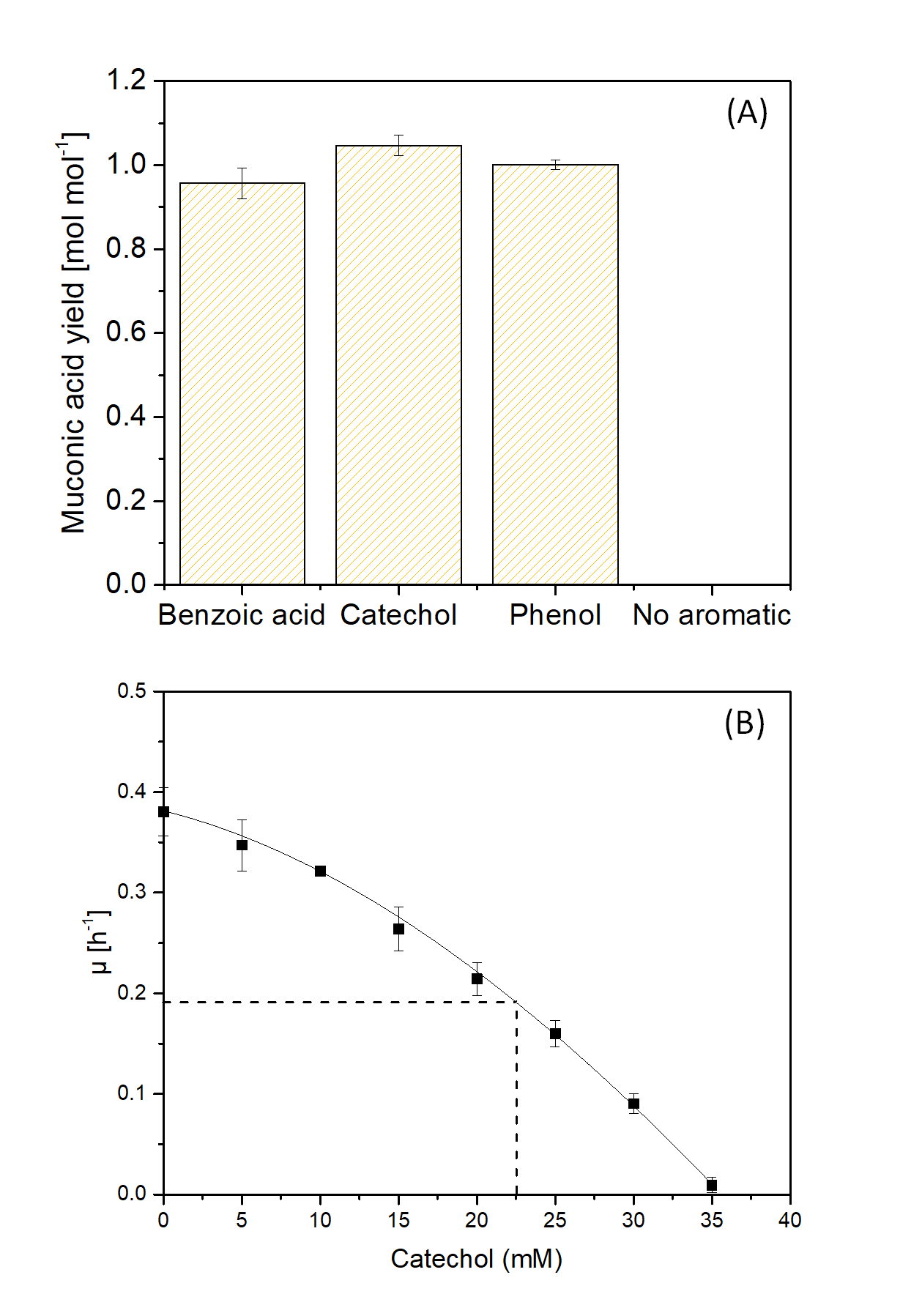
**

**Figure S2:** Growth and *cis-cis*-muconic acid (MA) production from small aromatics, using *Corynebacterium glutamicum* MA-1. The yield for MA on benzoic acid (20 mM), catechol (10 mM), and phenol (5 mM) was obtained from measurement of substrates and product at the beginning and after 24 hours of incubation (A). The tolerance to catechol was obtained from cell growth measurement (B). All data represent mean values and standard deviations from three biological replicates.


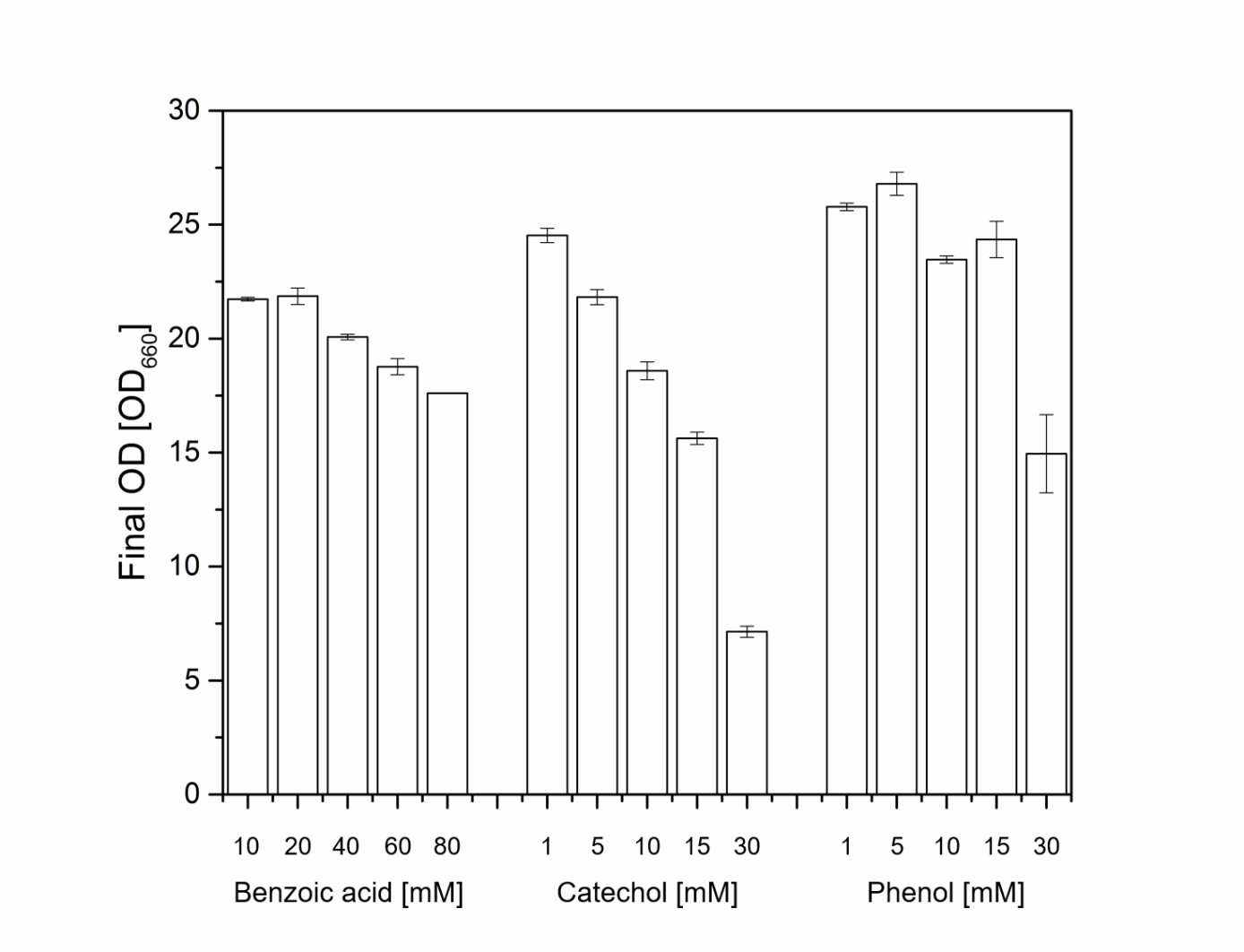


**Figure S3:** Tolerance of *Corynebacterium glutamicum* MA-1 against benzoic acid (A), catechol (B), and phenol (C). In addition, glucose was added as growth substrate. The final cell concentration was measured after 24 hours of cultivation. The data represent mean values and standard deviations from three biological replicates.


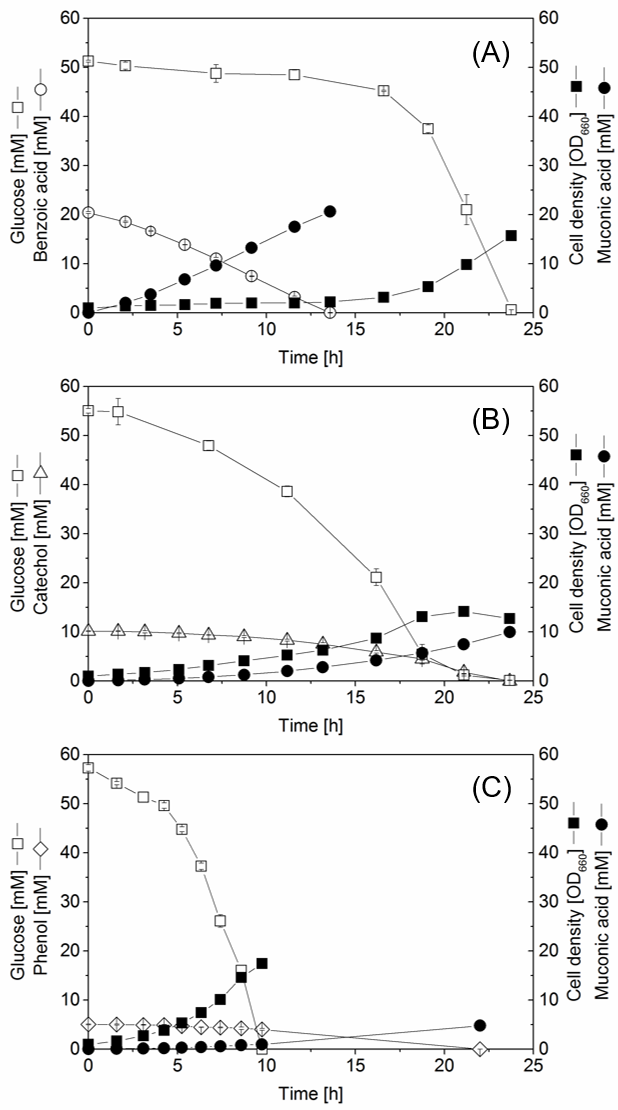


Figure S4: Kinetics and stoichiometry of *cis-cis*-muconic acid (MA) production, using the first generation producer *Corynebacterium glutamicum* MA-1. The aromatics benzoic acid (A), catechol (B) and phenol (C) were used for production. In addition, glucose was added as growth substrate. The data represent mean values and standard deviations from three biological replicates.

**
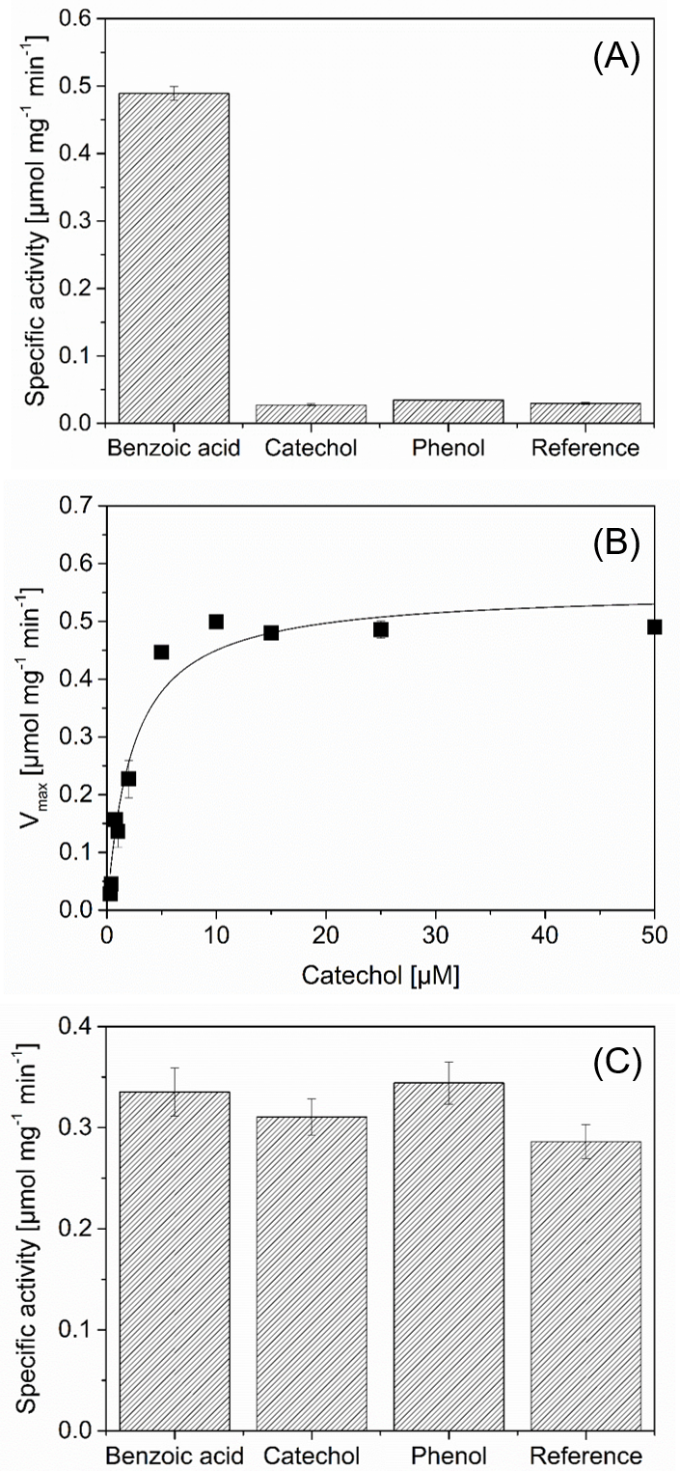
**

**Figure S5:** Characteristics of catechol-1,2-dioxygenase (CatA) in *Corynebacterium glutamicum*, grown on different aromatics. The data comprise the specific enzyme activity of the first generation producer *C. glutamicum* MA-1 (A), the kinetics of the enzyme with a fit of the experimental data from benzoate-grown cells to a Michaelis-Menten type kinetics (B), and the specific enzyme activity of the second generation producer *C. glutamicum* MA-2 (C). The data represent mean values and standard deviations from three biological replicates.


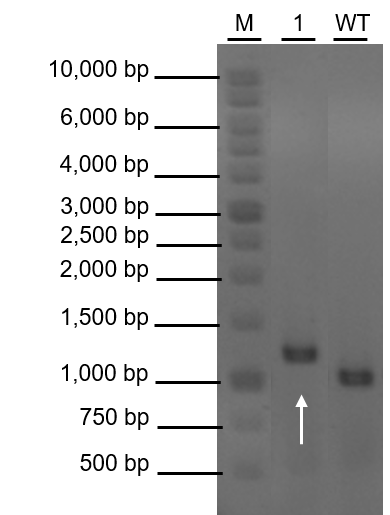


Figure S6: Confirmation of overexpression of the *cat*A gene under control of the *tuf* promoter in *Corynebacterium glutamicum* MA-2, using colony PCR. To this end, *C. glutamicum* MA-1 had been transformed with the integrative plasmid pClik int *sacB P_tuf_catA*, followed by recombination and selection. The primers P*_ef-tu_-catA* TS1 FW and P*_ef-tu_-catA* TS2 RV were used for the PCR (Table 1). The positive clone, indicated by the white arrow, revealed the increased fragment size, expected for the promoter exchange. M, 1 kb DNA ladder; 1, positive clone; WT, wild type.


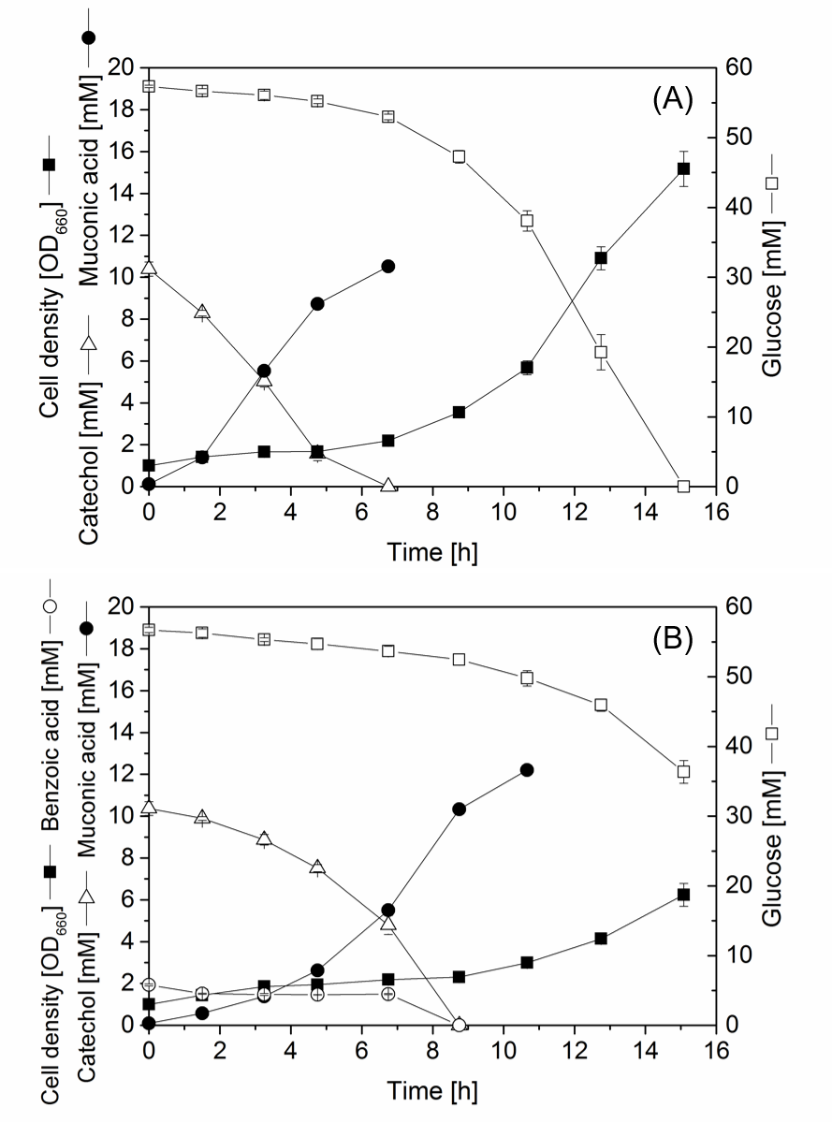


Figure S7: Kinetics and stoichiometry of *cis-cis*-muconic acid (MA) production, using the second generation producer *Corynebacterium glutamicum* MA-2 on catechol (A) and the first generation producer *C. glutamicum* MA-1 on catechol and benzoic acid (B). Glucose was added as growth substrate. The data represent mean values and standard deviations from three biological replicates.

**
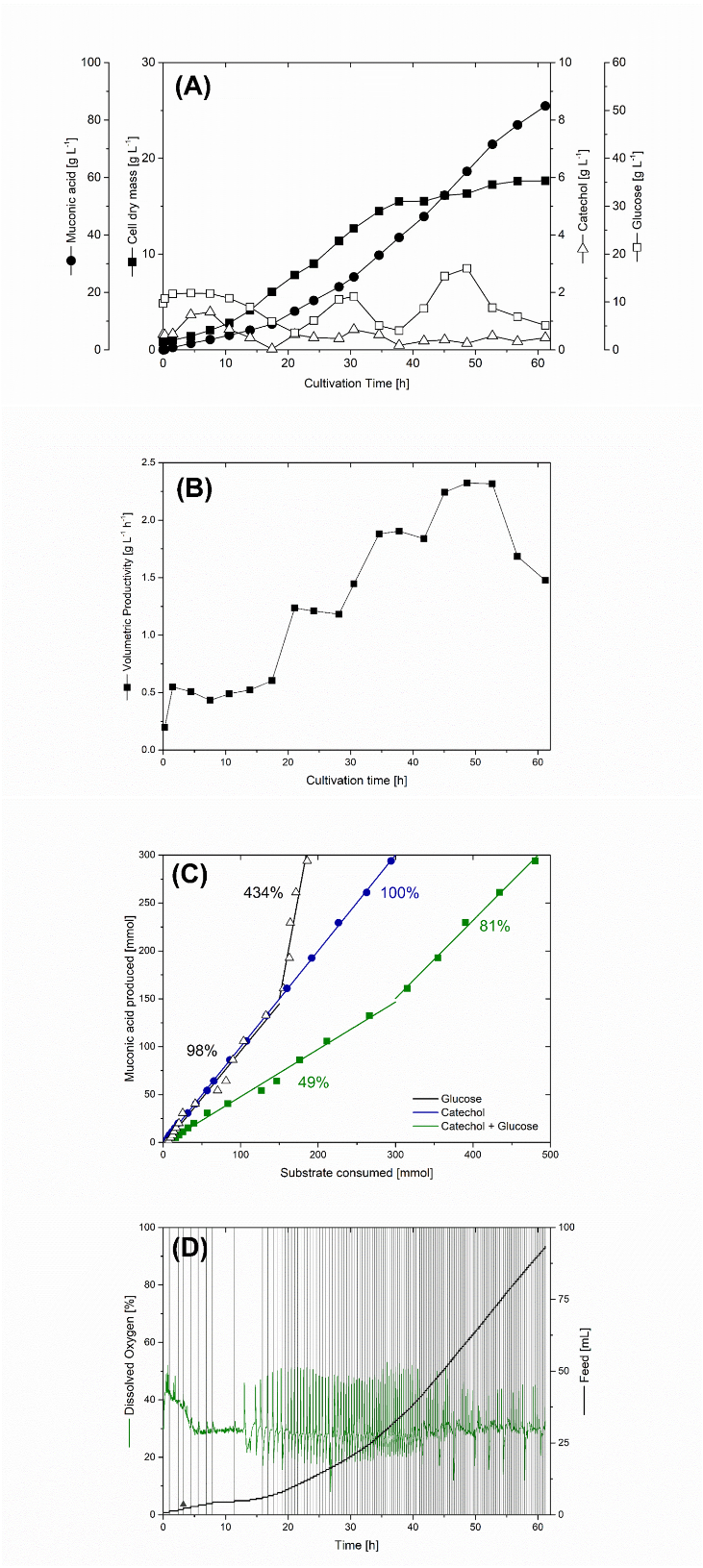
**

**Figure S8:** Fed-batch production of c*is-cis*-muconic acid (MA) from catechol by metabolically engineered *Corynebacterium glutamicum* MA-2. Substrate consumption, growth and MA formation (A). Volumetric productivity (B). Yields for MA from catechol, and for MA from catechol plus glucose (C). Pulse-wise feeding of catechol (D). Glucose was added continuously to maintain the glucose level in the range between about 5 to 15 g L^-1^ (A). The vertical lines represent individual catechol feed pulses (D). The feed frequency was variably adjusted, depending on the signal of dissolved oxygen, which precisely indicated the time point of catechol depletion. As example, feeding was halted once during the initial phase, corresponding to transient catechol accumulation and was accelerated later in response to the faster conversion. The data represent mean values and deviations from two replicates.
